# The Relationship Between Engagement Time in Case-based Learning and Performance on Preclinical Medical Education Exams

**DOI:** 10.1007/s40670-024-02112-x

**Published:** 2024-07-13

**Authors:** Ashley M. Tuin, Thomas Schechter, Cassie A. H. Eno

**Affiliations:** Creighton University School of Medicine, Omaha, NE USA

**Keywords:** Medical education, Assessment, Case-based learning, Team-based learning, Active learning

## Abstract

Case- and team-based learning activities are increasingly popular in medical education. Our institution utilizes a novel approach of using case-based learning (CBL) as prework for team-based learning (TBL) in preclinical medical education. This study evaluated the relationship between the time spent in CBL and performance on subsequent assessments including TBL performance on the IRAT and TRAT, and exam performance. Positive relationships were found between IRAT and exam performance. Time spent completing CBL was not found to be related to IRAT or exam performance. Implications for medical education are discussed.

## Background

Many medical schools in the past 20 years have integrated team-based learning (TBL) into their medical curriculum [1–5]. In fact, the AAMC reported that approximately 54% of medical schools utilized TBL in the 2019–2020 academic year [6]. Historically, the TBL structure is as follows: pre-class preparation, individual readiness assurance test (IRAT), team readiness assurance test (TRAT), immediate feedback/clarification, and clinical problem-solving activities [7]. The pre-class preparation typically involves the use of pre-reading or online lectures as prework for the TBL in the medical curriculum [7, 8]. The use of TBL in the preclinical years of medical education has been shown to provide many benefits including improved performance on exams and positive student feedback [1, 2, 8, 9]. A study from Carrasco et al. concluded “team-based learning scores and participation are better predictors of successful course performance than case-based learning performance” [2].

The AAMC reported that approximately 97% of medical schools utilized case-based learning (CBL) in the 2019–2020 academic year [6]. Though an extremely popular method of medical education, the use of CBL as preparation for TBL is less widely used and largely unstudied [2, 3, 7]. Creighton University School of Medicine (CUSOM) has modified the typical structure to use CBL as prework and preparation for TBL instead of the traditional TBL pre-reading or online recordings [7, 8]. Students at CUSOM first work through the clinical aspects of a case with their small group in CBL. Then, students attend a TBL based on the material presented in the CBL. Students are tested individually and as part of their team/small group. In this approach, TBL assesses the material that students learn with their small group in CBL, leading us to question the relationships between aspects of learning in CBL and performance in TBL. Though students at CUSOM spend the majority of time learning from traditional lectures, students complete one to three TBL’s per week, making up a significant portion of their instruction.

Guidelines for TBL are published in the student handbook and generated by the Office of Medical Education. This guideline, however, does not contain any requirements or expectations regarding CBL as preparation for TBL, leaving small groups with free direction to complete the case as they wish at a pace set by the group. Additionally, there is no graded component tied to the completion of the CBL. Aside from the first four cases of the M1 year, there is no direct faculty participation or oversight in the small group setting. Given the lack of direct oversight, there may be a tendency for groups to rush through or sidestep difficult aspects of the case to complete the task more quickly, significantly hindering both the individual student’s and group’s learning.

Given CUSOM’s novel approach of using CBL in preparation for TBL, we aimed to evaluate the relationship between the time spent in CBL, performance on the TBL, including IRAT and TRAT scores, and performance on final exam questions assessing the content of the CBL.

## Activity

Student data from two medical campuses of CUSOM were collated to examine the relationship of the identified variables from 29 cases across 6 courses throughout the academic year 2022–2023 that met inclusion criteria as discussed below. Data from the Omaha campus included the entire M2 cohort (class of 2025) that consisted of 128 students in the M2 year. Data from the Phoenix campus included the entire M2 cohort (class of 2025) that consisted of 99 students in the M2 year. The following variables were collected: time spent in CBL (in minutes; extracted from the learning management system), IRAT and TRAT performance (extracted from Intedashbaord), and exam performance on related questions (extracted from ExamSoft). Of note, exam performance refers only to final exam questions that tested material directly related to CBL/TBL sessions, not to overall final exam performance in the course. CBL cases are available through the learning management system which requires students to click through the case in order to reach the next step of the case. Once the team has completed the case, a student moderator must hit submit in order to see the correct answers for questions that were completed throughout the case. With the submission of the case, completion time is recorded. Data was merged by student ID and then de-identified. Case times were calculated by subtracting the start time recorded from the completion time recorded. Outliers with respect to time (longer than 160 min for a scheduled 120-min time slot) were removed. Only cases with associated exam questions were included in analysis. A mean score for each variable across all cases was computed for each student, taking the final score from each relevant case and finding the mean average across all cases for IRAT, TRAT, and exam data. Data was transformed through centering and standardization, resulting in *z*-score data for each observation of each variable to allow for easier interpretation of comparisons. *Z*-scores were chosen for the variables due to the ease of interpretation with all variables being on the same scale, save for exam scores, which were kept on a 0 to 1 scale as provided in the data; *Z*-scores also allow for results of regression modeling to be interpreted in standard deviations. Computing variable values to *z*-scores also prevents additional weight from being assigned to variables on different scales.

## Results and Discussion

Linear regression was performed on the resulting data for relationships between time spent on the CBL, IRAT performance, TRAT performance, and exam performance. Analysis on the distribution of residuals, the difference between actual and predicted observations, was done to validate model performance on the data. All analysis was carried out in R, with RStudio serving as the integrated development environment.

### Omaha Results

**Fig. 1:**
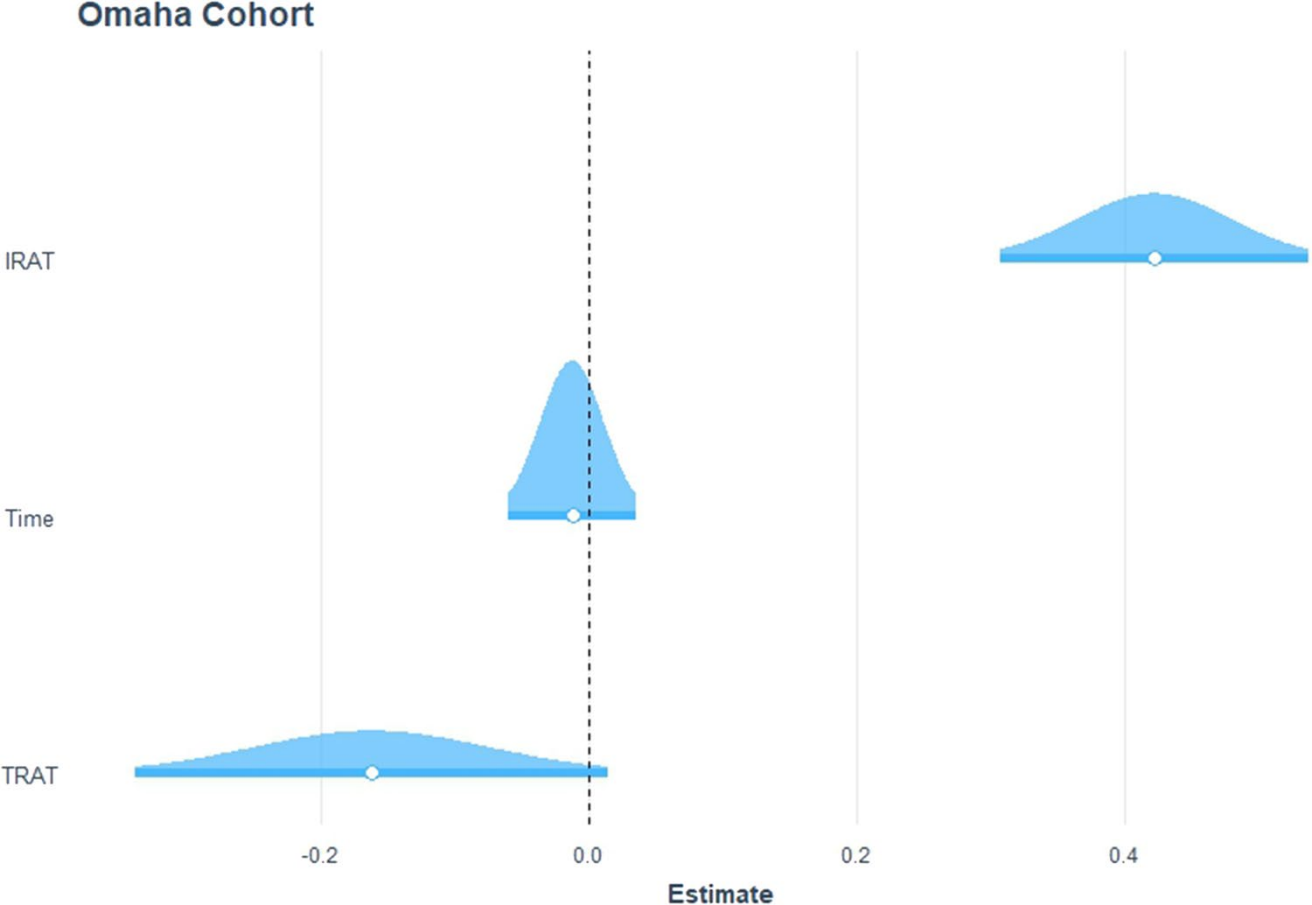
Distribution of estimated effect sizes from the chosen model

**Fig. 2:**
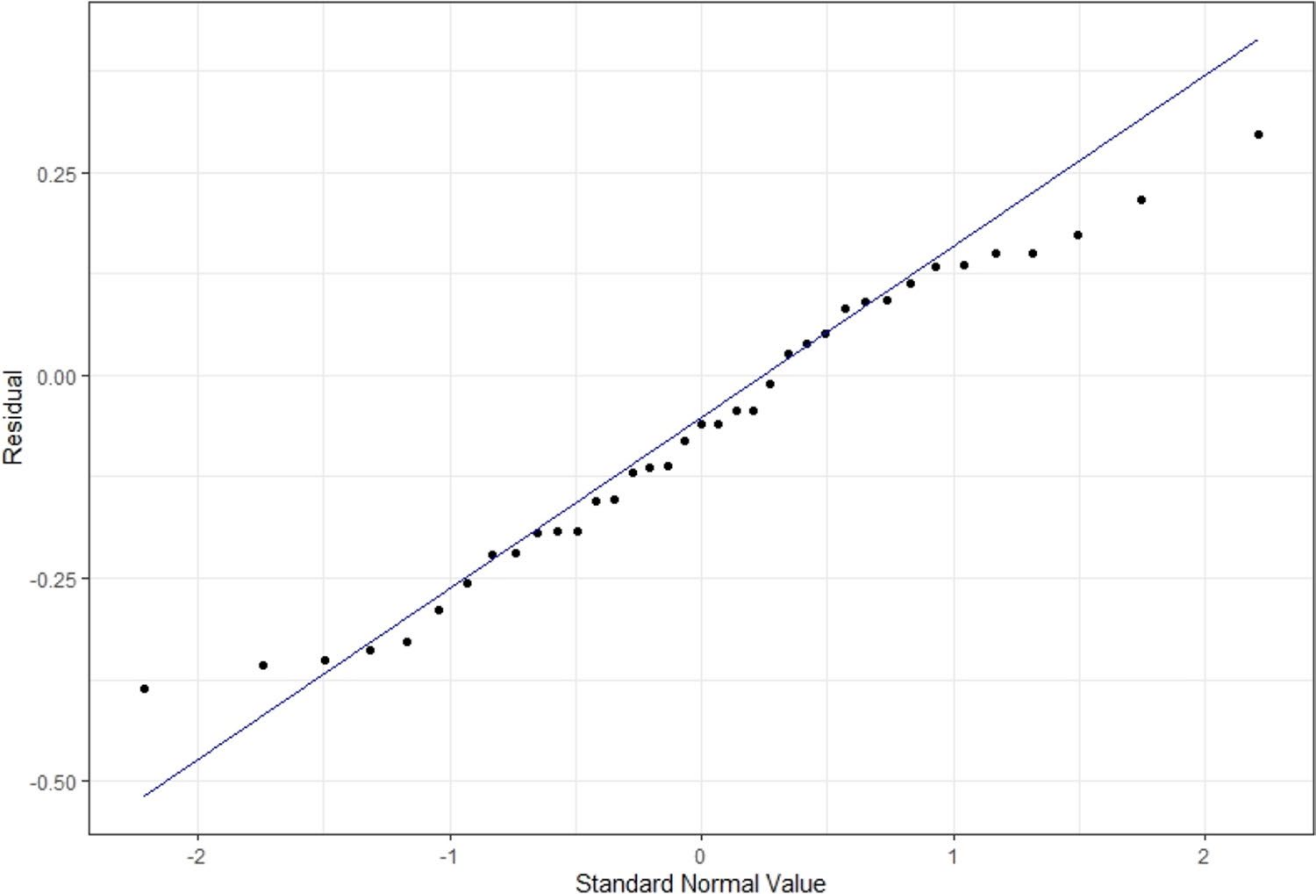
Approximate normal distribution of residuals for the chosen model, with the rightward skew indicated by the points above the trend line on the lower left and below the trend line on the upper right portion of the graph

There was a significant relationship between IRAT and exam performance, controlling for time and TRAT scores, with exam performance as the dependent variable (adj. *R*^2^ = 0.42, *F*(6, 73) = 10.3, *p* = 0.00). The coefficient from this model was *β* = 0.40, indicating that a 1 standard deviation increase in IRAT score would lead to an approximately 0.40 standard deviation increase in exam score (Fig. 1). Predictions of these exam performances resulted in a distribution of residuals with a rightward skew, indicating a higher-than-observed estimation by the model for exam scores based on IRAT scores and controlling for time and TRAT scores (Fig. 2). This data supports previous research showing a strong correlation between TBL performance and final exam performance when compared to other learning methods [1, 2].

There was not a significant relationship between time and exam performance (adj. *R*^2^ = 0.00, *F*(1, 78) = 1.02, *p* = 0.317). There was not a significant relationship between average time and average IRAT performance (adj. *R*^2^ = 0.00, *F*(1, 78) = 1.07, *p* = 0.30). There was not a significant relationship between average time and average TRAT performance (adj. *R*^2^ = 0.00, *F*(1, 78) = 13.12, *p* = 0.71). This indicates that exam performance, average IRAT performance, and average TRAT performance cannot be predicted by the average time spent on the case.

###  Phoenix Results

**Fig. 3:**
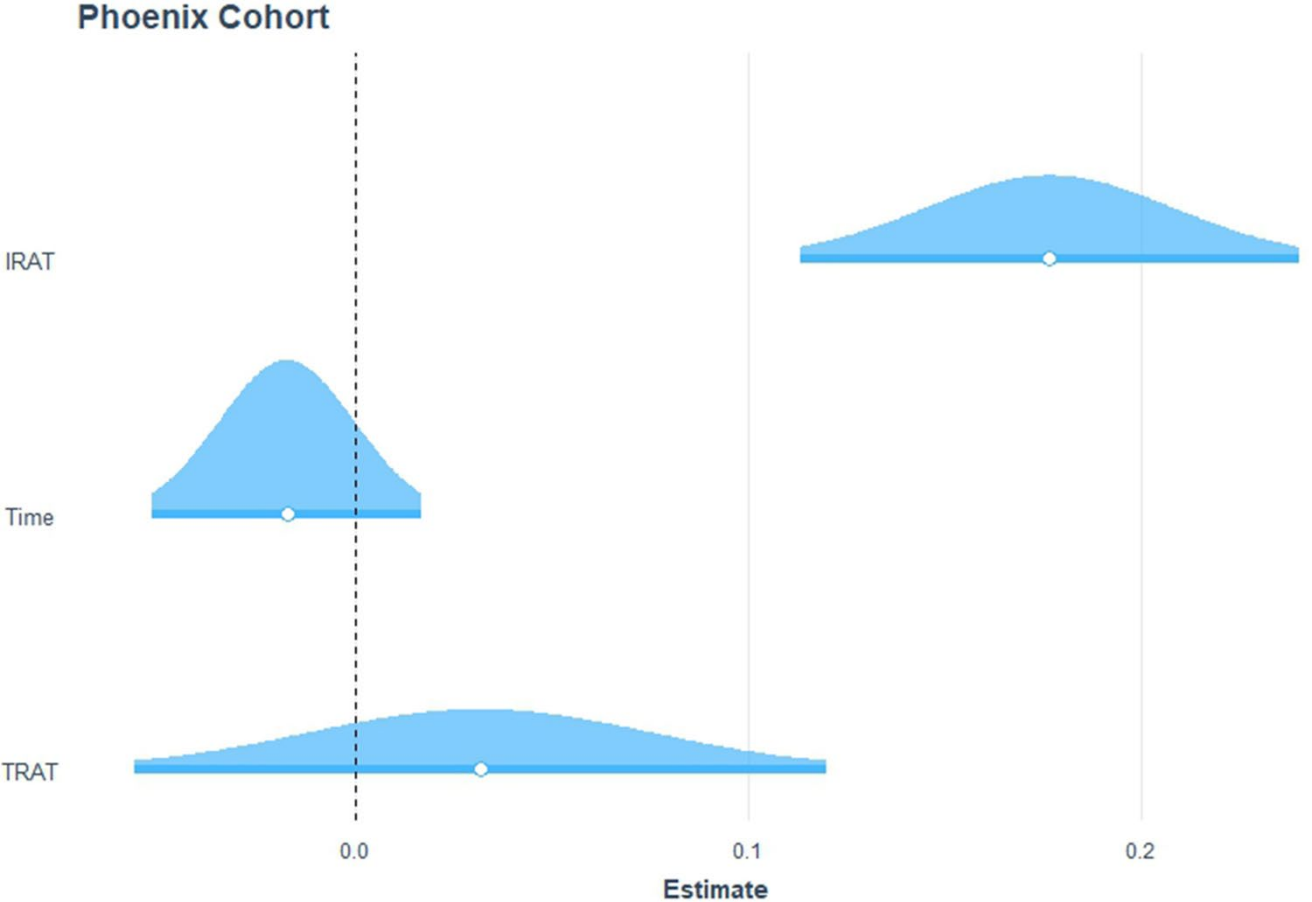
Distribution of estimated effects sizes from the chosen model

**Fig. 4:**
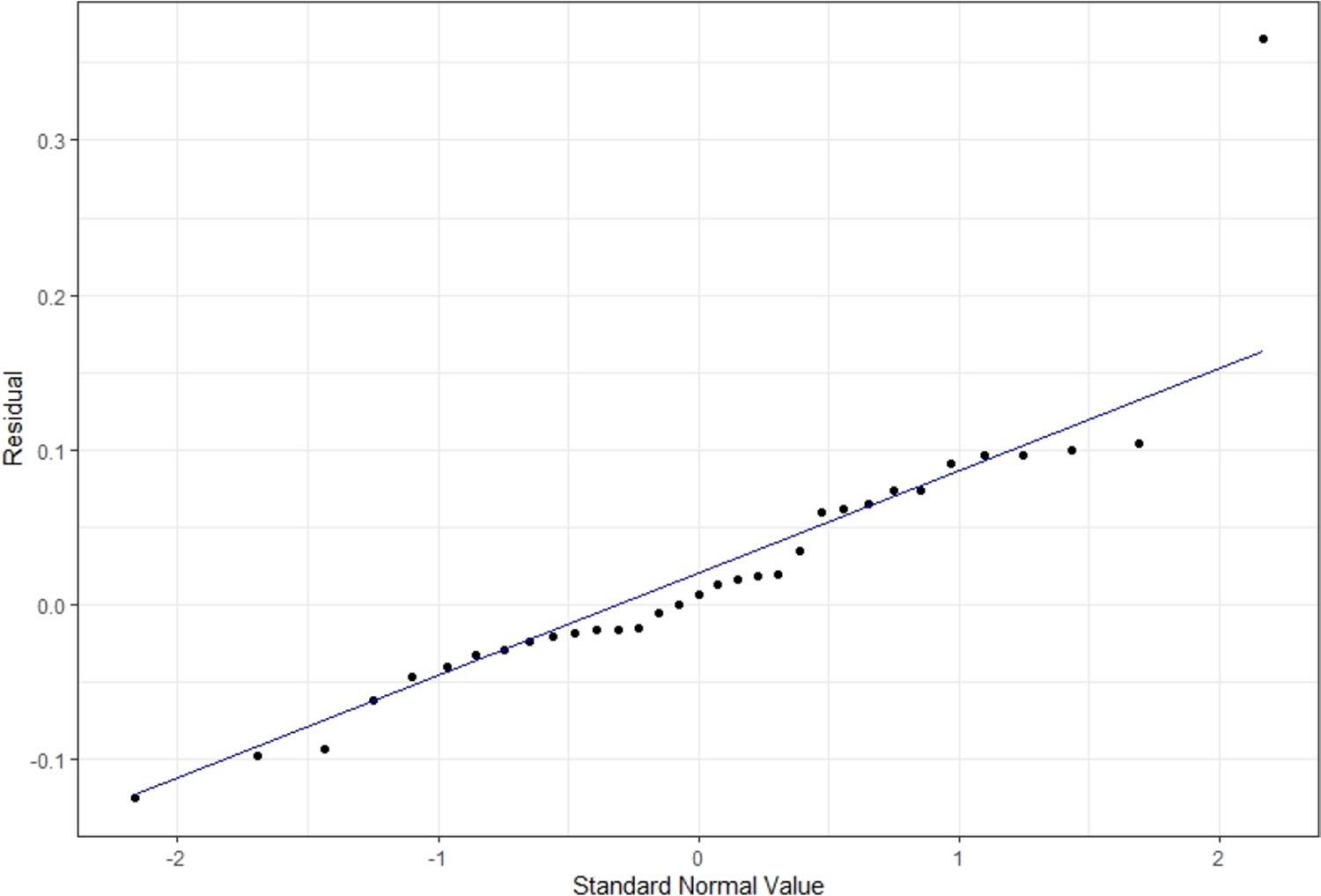
Non-normal distribution of residuals for the chosen model, shown by small systematic deviations near center of distribution. An outlier exists in the rightward tail of the distribution

There was a significant relationship between average IRAT and average exam performance, controlling for time and mean TRAT scores, with exam as the dependent variable (adj. *R*^2^ = 0.33, *F*(3, 70) = 13.12, *p* < 0.01). The coefficient from the model was *β* = 0.18, indicating that a 1 standard deviation increase in mean IRAT score would lead to an approximately 0.18 standard deviation increase in mean exam score (Fig. 3). Predictions of these exam performances resulted in a non-normal distribution of residuals, indicating that not all patterns within the data between mean IRAT scores and mean exam scores, when controlling for time and mean TRAT performance, were captured by the model. Figure 4 also indicates that there may exist outliers in the tail end of the residual distribution.

There was not a significant relationship between average time and exam performance (adj. *R*^2^ = 0.00, *F*(1, 72) = 0.46, *p* = 0.50). There was not a significant relationship between IRAT average performance and average time (adj. *R*^2^ = 0.00, *F*(1, 105) = 0.46, *p* = 0.78). There was a significant relationship between TRAT average performance and average time (adj. *R*^2^ = 0.03, *F*(1, 105) = 4.25, *p* = 0.04). However, a low coefficient of determination indicates that very little variation in average TRAT scores can be explained by average time. These results indicate that exam performance and average IRAT and TRAT performance cannot be predicted by average time spent on the case.

Similar results were found in Omaha and Phoenix with respect to average IRAT performance being significantly related to exam performance, with a larger effect on exam performance on the Omaha campus. Average TRAT performance was not significantly related to average time in Omaha, but it was in Phoenix albeit a low coefficient of determination. There was not a significant relationship between IRAT results and average time on either campus.

Though previous research has found a relationship between TBL participation and IRAT scores, participation was defined as the amount of time a student spoke during the activity [2]. Given CUSOM’s use of CBL as pre-work for TBL, we considered time spent in CBL as an alternative variable that may be comparable to participation in the classical TBL model. Though student participation and time spent in CBL cannot be directly equated, it is assumed that the longer a group spends completing CBL, the greater the amount of participation of the students in the group. Therefore, we expected to see a relationship between performance and time spent in CBL. However, our data demonstrated no relationship between time spent on CBL and individual performance on TBL and exams. Therefore, it cannot be assumed that case time will act as a strong predictor of assessment performance.

Faculty and educators at CUSOM have made efforts to improve the quality and experience of the CBL’s, often focusing on the time and experience of the CBL. This research indicates that increasing time spent in the CBL may not have an impact on students. Our data suggests that there is no correlation between time spent completing CBL and performance on IRAT and exam questions. Though a relationship was found between time spent completing CBL and performance on TRAT at the Phoenix campus, it explains very little of the variation in TRAT scores. This would indicate that setting protocols relating to time may not provide any direct benefit to student academic performance. Future research should consider the time students spend studying outside of CBL and TBL. It is possible that students who move through the CBL at a faster pace spend more time individually studying as preparation for TBL and, therefore, perform just as well as groups who engaged in more discussion throughout CBL. Future research could help to refine the understanding of the relationship between time and performance.

This study was conducted at a single institution. While this limits generalizability, this research also provides a framework for future research in this area. Some case times were missing from the data; *z*-score mean imputation was used to counter this issue. Results are limited to the group time submitted in the CBL, and it is possible that some individuals would have spent more or less time engaged with the material. There are many other aspects to CBL and TBL that may also influence performance, which could not be studied in this particular study, including but not limited to, student background knowledge, English as a second language, student participation in both CBL and TBL, and preferred learning method.

Overall, our findings suggest that there is a relationship between performance on TBL and exam performance. Our data supports previous literature indicating better course performance stemming from these active learning methods [1, 2, 9]. We found no statistical relationship for time spent on CBL and related assessment performance, which may provide valuable feedback to medical educators on the use of CBL and TBL in preclinical medical education.

## Data Availability

The datasets generated for the current study are not publicly available but are available from the corresponding author on reasonable request.
